# Identification of the toxin components of *Rhizoctonia solani* AG1-IA and its destructive effect on plant cell membrane structure

**DOI:** 10.3389/fpls.2024.1348257

**Published:** 2024-02-13

**Authors:** Shanshan Xu, Shaofeng Ren, Wenjing Bao, Xiaoguang Li, Yumei Zhang, Buzhu Yu, Weiqi Li, Chengyun Li, Wenhan Dong, Genhua Yang

**Affiliations:** ^1^ State Key Laboratory for Protection and Utilization of Bio-Resources in Yunnan, Yunnan Agricultural University, Kunming, Yunnan, China; ^2^ School of Pharmacy, Guizhou University of Traditional Chinese Medicine, Guiyang, Guizhou, China; ^3^ Xishuangbanna Tropical Botanical Garden, Chinese Academy of Sciences, Xishuangbanna, Yunnan, China; ^4^ Institute of Botany, Chinese Academy of Sciences, Kunming, Yunnan, China

**Keywords:** *R. solani* AG1-IA, rice sheath blight, toxin, membrane components, membrane structure

## Abstract

Rice sheath blight is a fungal disease caused mainly by *Rhizoctonia solani* AG1-IA. Toxins are a major pathogenic factor of *R. solani*, and some studies have reported their toxin components; however, there is no unified conclusion. In this study, we reported the toxin components and their targets that play a role in *R. solani* AG1-IA. First, toxins produced by *R. solani* AG1-IA were examined. Several important phytotoxins, including benzoic acid (BZA), 5-hydroxymethyl-2-furanic aid (HFA), and catechol (CAT), were identified by comparative analysis of secondary metabolites from AG1-IA, AG1-IB, and healthy rice. Follow-up studies have shown that the toxin components of this fungus can rapidly disintegrate the biofilm structure while maintaining the content of host plant membrane components, thereby affecting the organelles, which may also explain the lack of varieties highly resistant to sheath blight.

## 1 Introduction

*Rhizoctonia solani* has a wide range of hosts and strong saprophytic activity. This fungus can cause major harm to thousands of plants, including plants in the Leguminosae, Cruciferae, Solanaceae, and Gramineae families, and can lower the quantity and quality of crops such as rice, corn, and potatoes (Farr and Rossman, 2021). Rice sheath blight is an important rice disease that occurs in all rice-producing areas of the world and has become the most prevalent major disease of rice in many rice-producing areas (Chen et al., 2011). To date, high-resistance sources have not been found in the germplasm bank, so it has been impossible to cultivate new materials resistant to *R. solani* through transgenic methods (Xu et al., 2020), and the study of the pathogenic mechanism of *R. solani* can provide targets for disease resistance breeding.

Toxins are important disease-causing factors that cause a high infection rate and severe damage to the host via a very complex pathogenic mechanism. It has been demonstrated that toxins can affect multiple organelles. For example, a toxin produced by the black-spot pathogen of *Zizyphus jujuba* cv. Dongzao increases cell membrane permeability in the host plant, leads to leakage of cell electrolytes, and leads to damage of the membrane system and disruption metabolism, thus causing plants to lose their normal physiological functions (Yu et al., 2008). When tabtoxin comes into contact with tobacco leaves, the toxin damages the inner chloroplast membrane system of tobacco leaves, causes the disintegration of the basal lamellae of tobacco leaves, and leads to the formation of vesicles in leaf chloroplasts, which is a serious toxic effect on tobacco plants (Xu and Zhang, 2006). When rice healing tissues are treated with toxins produced by *M. oryzae*, blurring of the bilayer membrane and cristae, vacuolization of mitochondria, and plasma membrane rupture are observed (Wei et al., 2000). In 2007, Brooks (2007) developed a method to isolate and utilize a phytotoxin from *R. solani* to assess its genetic susceptibility to rice sheath blight. Vidhyasekaran et al. (1997) and Chen et al. (2009) reported significant electrolyte extravasation of the toxin in rice tissues treated with the toxin, and the longer the treatment time was, the greater the concentration of the toxin and the greater the extravasation rate. This is because the toxin acts on the biofilms composed of lipids and proteins, which alters cell membrane permeability and electrolyte extravasation. However, *R. solani* toxins have not been clearly defined and are mostly considered carboxylic acids and derivatives (Aoki and Sassa, 1963; Chen et al., 2011; Yang et al., 2011; Kankam et al., 2016a; Hu et al., 2018) or sugars and glycosaminoglycans (Vidhyasekaran et al., 1997; Sriram et al., 2000). There has also been no research on the impact of its single compound on the host plant.

*R. solani* AG1-IA is the main pathogen of rice sheath blight. Although the main components of *R. solani* toxins have been studied, the conclusions differ. In our previous study (Yang et al., 2008), we isolated the AG1-IB subgroup from rice, and while AG1-IA was more pathogenic than AG1-IB, both of these strains belonged to the AG1 anastomosis group. In this study, we compared the compositions of the crude extracts of the AG1-IA and AG1-IB strains. In addition, we screened for toxin components associated with pathogenicity in AG1-IA and detected three compounds in crude extracts that damage plant cell membranes and organelles, which provide a basis for the subsequent study of the pathogenic mechanism of AG1-IA, differences in pathogenicity between IA and IB strains, and the key factors involved in virulence specificity.

## 2 Materials and methods

### 2.1 Determination of strain pathogenicity

The rice variety used in this study was Yunnan local indica conventional rice variety: Bozhugu, which was obtained from Yunnan Agricultural University (Yunnan, China). The *R. solani* test strains used were AG1-IA M-9-11, M-03-14, M-37, MV-5, and AG1-IB: HX-4C, HX-40, HX-48, and X-4-1-8, respectively (Zhang, 2016). The preserved test strains were activated and inoculated on Potato Dextrose Agar (PDA) solid medium and cultured at room temperature (25 ± 5°C) until the hyphae covered the medium and produced sclerotia. The medium with hyphae and the wet absorbent cotton was wrapped in the sheath of the third leaf sheath of the rice plant at the late tillering stage. The temperature was 28 ± 2°C, and the humidity was above 90%. Each treatment was repeated five times, and the blank medium was used as the control to calculate the relative lesion height after 10 days.


Relative lesion height % (RLH%)=measured lesion height/plant height×100

### 2.2 Activity detection of crude extract

The AG1-IA M-9-11 and AG-1 IB HX-4C strains preserved on barley grain were subsequently transferred onto PDA solid medium and incubated at 25°C for 7 days. After the mycelium had grown throughout the dish and produced sclerotia, 6-mm blocks were transferred to a new PDA solid medium and incubated at 25°C for 2, 4, or 6 days. After cutting the PDA medium and subjecting the portion to three ethyl acetate extractions, the ethyl acetate phases were combined, evaporated, and concentrated to dryness at 45°C to obtain a yellowish-brown viscous extract of the crude toxin. Then, 0.1 g of crude extract was added to 1 mL of sterile water and injected into lettuce and rice leaves to observe its effects.

### 2.3 Analysis of crude extract components

To identify the components of the crude extract of AG1-IA, we used the weakly virulent AG1-IB strain and healthy rice as controls for detection via GC-MS. Activated strains (AG1-IA M-9-11 and AG1-IB HX-4C) were cultured on PDA medium at 25°C for 7 days. Five 6-mm-diameter blocks were transferred to the Richard liquid medium and incubated at 28°C for 18 days. The filtrate was collected and concentrated to 1/10 volume at 60°C under reduced pressure with a rotary evaporator, extracted three times with ethyl acetate, and evaporated to dryness at 45°C to obtain the crude extract. The crude extract was dissolved in ethyl acetate, filtered by needle filter filtration, and collected in 1.5-mL brown sample bottle tubes. The GC conditions were described in detail previously (Gao et al., 2018).

The crude extract of AG1-IA was mixed with 80–100 mesh silica gel at a ratio of 1:1, and subsequently placed on a silica gel column. Petroleum ether/ethyl acetate (6:1–0:1) was used for gradient elution. Each fraction was subjected to thin-layer chromatography (TLC). After the development layer was colored, the activities of the separated components were determined, and repeated silica gel column chromatography was performed. Gradient elution was carried out with chloroform/methanol (30:1–6:1). The fraction was collected, and the related activity of each fraction was determined. The active fraction was eluted on a Sephadex LH-20 column with a chloroform/methanol (1:1) gradient. Similarly, the activity of each eluent fraction was determined by high-performance liquid chromatography (HPLC). The eluent was methanol/water/glacial acetic acid (10:90:1). The separated and purified compounds were analyzed via NMR, which was completed by the Instrument Sharing Center of Kunming Institute of Botany, Chinese Academy of Sciences.

### 2.4 HPLC verification and activity determination of three compounds

AG1-IA M-9-11, AG1-IB HX-4C, and lettuce leaves inoculated with M-9-11 2 days after activation growth were soaked in methanol three times. The methanolic extract for each group was combined, and methanol was evaporated with a rotary evaporator at 35°C. After the extract was dissolved in water, ethyl acetate was used for extract three times, and the ethyl acetate extract was combined, followed by ethyl acetate evaporation with a rotary evaporator at 40°C. The extract was dissolved with an appropriate amount of methanol and subjected to HPLC analysis. The crude extract extracted from the strain fermentation broth and the standards for each compound were used as control. The chromatographic conditions were as follows: gradient elution with methanol:water (containing 0.1% glacial acetic acid) from 5% to 95% for 40 min and a flow rate of 0.8 mL/min.

Based on the GC-MS and HPLC data and the concentrations of standards, the concentration of each compound in the fermentation broth was initially determined, and different concentrations of the compound solutions were subsequently prepared; first, 200 μL of solution was added to a 2-mL centrifuge tube, and healthy rice leaves were cut and immersed in this solution. Leaf symptoms were observed after 24 h. In the absence of symptoms, the concentration of each compound increased. Otherwise, the concentration of the compound was reduced.

### 2.5 Effects of crude extract on membrane lipid components

*Arabidopsis thaliana* and *Brassica napus* leaves treated with AG1-IA crude extract were subjected to lipid determination via an electrospray tandem mass spectrometry (ESI MS/MS)-based plant lipidomic method (Welti et al., 2002). Data analysis was performed with Excel and Origin 8.0.

### 2.6 Evaluation of the effects of crude extract on plant cell membrane integrity

Quantitative determination of the crude extract effects was performed via the phosphorus extravasation method (Chen et al., 2003). In the control group, 3 mL of blank medium extract solution was used, and 3 mL of the toxin solution was added to the treatment group. In total, 0.10 g of rice or lettuce leaves was added, shaken lightly, submerged in the treatment solution, and incubated for 24 h at 100 rpm/min in a constant-temperature shaker at 25°C. In total, 2 mL of the soaking solution was centrifuged at 10,000 rpm/min for 10 min. Then, 0.4 mL of the supernatant was collected. Subsequently, 2 mL of ammonium molybdate solution, 1 mL of sodium sulfite solution, 1 mL of hydroquinone solution, and 10 mL of distilled water were added, and the mixture was incubated for 30 min. The phosphorus content in each sample was determined from the measured absorbance, and each treatment was conducted in triplicate.

Fresh, healthy lettuce leaves were prepared into multiple discs of approximately 15 mm in diameter using a hole puncher, and the leaves were arranged in a Petri dish lined with moist filter paper. In total, 10 μL of crude extract was added, and the mixture was subsequently mixed well with a cotton swab. Five replicates of each treatment were performed, and the plants in the control group were left untreated. The treated leaves were placed in the dark for approximately 20 min, after which relevant photosynthetic parameters were measured with the chlorophyll fluorescence imager.

Protoplasts were obtained as described previously (Yoo et al., 2007). Twenty microliters of 1×10^6^ cells/mL protoplasts was pipetted into PCR tubes; 70 μL of 50×, 100×, 200×, or 500× crude extract was added; and the volume was adjusted to 100 μL. After 30 min or 60 min, 20 μL of the reaction solution was pipetted to assess the morphology of protoplasts. Finally, the proportion of protoplasts with a normal morphology was determined as follows.


$$\begin{array}{c} The\;proportion\;of\;normal-form\;protoplasts\\ =number\;of\;protoplasts\;with\;normal\;morphology/total\;number\;of\;protoplasts\times 100\% \end{array}$$

In total, 10 μL of vegetable oil and 5.99 mL of distilled water were pipetted into a 10-mL centrifuge tube and mixed into an emulsion by ultrasonication at 35 Hz and 30°C. The absorbance was measured at 600 nm. Then, the test compounds were mixed at each concentration, and the absorbance was measured after the sample had stood for 5 min, 20 min, 40 min, or 60 min. Finally, the rate of change was calculated based on the change in absorbance and treatment time.

After dilution of the crude extract 100 times (0.01 g/mL), 200 times, 500 times, and 5,000 times with water, 2 mL was added to a six-well plate, after which the cultured Arabidopsis plants were submerged in the crude toxin for 10 s, 30 s, 1 min, or 2 min. This was followed by transfer of the solution to 2 mL of sterilized water, washing off of the crude extract on the root surface, and transferring the mixture to PI dye solution (5 mg/mL). In total, 2 mL of sterilized water was used for washing after staining for 10 min (the controls were not treated but were directly placed in the PI dye solution for staining). Arabidopsis seedlings were placed on the slide, followed by mounting and analysis. Each treatment had 10 replicates, and the experiment was conducted in triplicate.

### 2.7 Effects of phytotoxins on cell structure and organelles

The test compounds were dissolved in water and prepared at different concentrations; first, 200 μL of each solution was added to a 2-mL centrifuge tube, and fresh healthy rice leaves (approximately 4 cm) were added. After a certain treatment time, the leaves were removed to prepare ultrathin sections, which were assessed with a transmission electron microscope (Fei TECNAI G2).

## 3 Results

### 3.1 Pathogenicity testing of AG-1 IA and AG-1 IB

**Figure 1:**
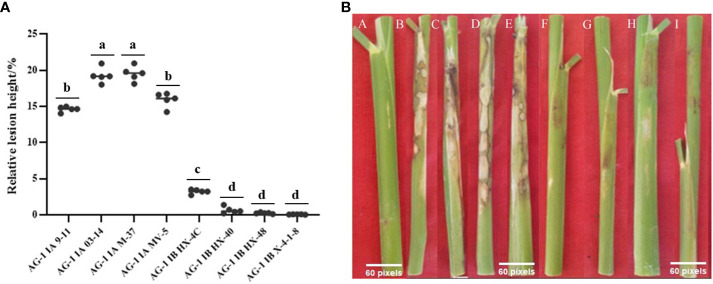
Results of rice relative lesion height method to determine the pathogenicity of *R. solani*. **(A)** The relative lesion height of different strains after inoculation with rice; each strain was measured in five replicates. Tukey’s HSD test (DPS v9.01) revealed pairwise significant differences at *p*< 0.05, represented by the lowercase letters. **(B)** Phenomenon after different strains were inoculated into rice plants; A: Control; B: AG1-IA M-9-11; C: AG1-IA M-03-14; D: AG1-IA M-37; E: AG1-IA MV-5; F: AG1-IB HX-4C; G: AG1-IB HX-40; H: AG1-IB HX-48; I: AG1-IB X-4-1-8.

The result (**Figure 1**) showed that the pathogenicity of the AG1-IA strains was significantly greater than that of the AG1-IB strains, and there were some differences in the pathogenicity of different strains in the same subgroup. Among them, the AG1-IA strain caused many cloudy lesions around the rice sheath, the M-37 strain had the strongest pathogenicity, and the M-9-11 strain had relatively weak pathogenicity. Within the IB subgroup of AG1, strain M-76 had the strongest pathogenicity, strain X-4-1-8 had the weakest pathogenicity, and there was almost no lesion at the inoculation site of the strain X-4-1-8 on the rice sheath.

### 3.2 Activity and duration of action assay for crude extracts

**Figure 2:**
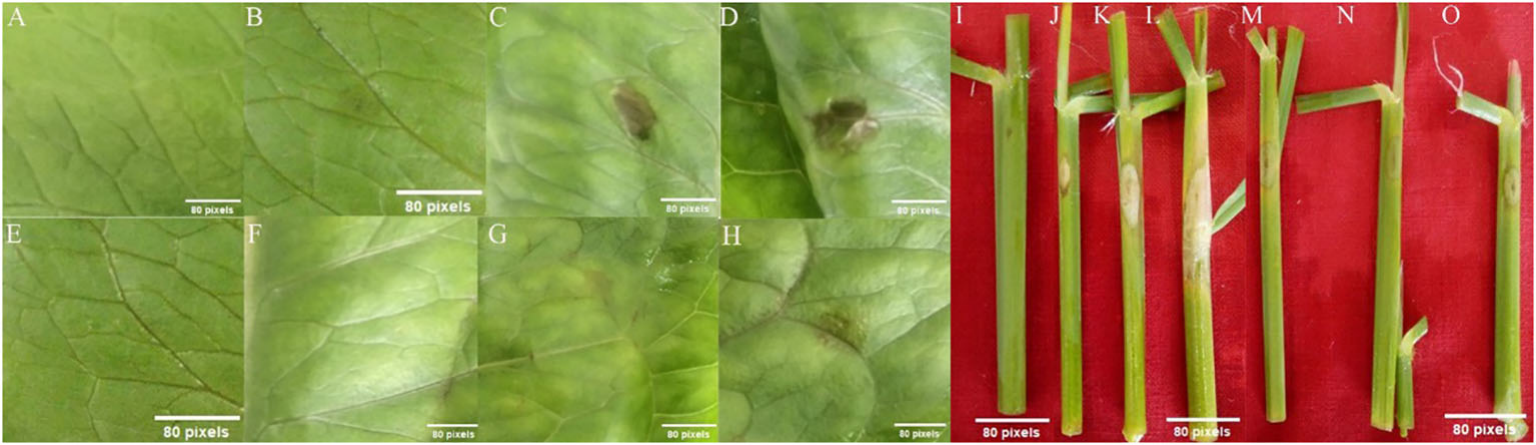
Symptoms of lettuce leaves treated with *R. solani* crude extract produced on PDA media. **(A, E, I)** Control; **(B, J)** pathogenicity of AG1-IA crude extract cultured for 2 days; **(C, K)** pathogenicity of AG1-IA crude extract cultured for 4 days; **(D, L)** pathogenicity of AG1-IA crude extract cultured for 6 days; **(F, M)** were treated with AG1-IB crude extract; **(G, N)** were treated with AG1-IB crude extract; **(H, O)** were treated with AG1-IB crude extract.

Five microliters of diluted crude extracts from *R. solani* AG1-IA M-9-11 and AG1-IB HX-4C cultured on PDA medium for 2, 4, and 6 days was injected into lettuce and rice leaves, respectively. The results showed that the crude extract could cause the formation of lesions similar to sheath blight on rice. In addition, rice and lettuce leaves developed mild necrosis due to the crude extract that was collected after 2 days of growth. After 6 days of culture, the crude extract of the strain was more pathogenic than was that obtained after 2 or 4 days, and AG1-IA was more pathogenic than AG1-IB. Additionally, the disease spot formed by the crude extract of AG1-IA was significantly larger than that formed by the crude extract of AG1-IB (**Figure 2**), which was consistent with the pathogenicity of the strain (**Figure 1**). The duration at which the crude extract acts on lettuce and rice leaves to produce lesions is consistent with the duration at which the strain acts to produce lesions (Doehlemann et al., 2009).

### 3.3 Component detection of crude extracts of AG1-IA and AG1-IB

Simultaneous production of spots by the crude extract and the pathogenic fungus was observed, so crude extract produced by pathogenic fungi may play an important role in the pathogenic process. Considering that the pathogenicity of AG1-IB is much lower than that of AG1-IA, we analyzed the crude extract of AG1-IA with AG1-IB and healthy rice as controls. Therefore, we tested the compositions of crude extracts and substances with peak matches greater than 85% in the AG1-IA and AG1-IB strains; healthy rice was also screened (**Supplementary Tables S1**–**S3**). The results showed that the volatile components in crude extract of AG1-IA were mainly esters, carboxylic acids, ketones, alkanes, phenols, alcohols, and amides. The relative contents of benzoic acid (BZA), 2,2′-methylenebis-6-(1,1-dimethylethyl)-4-methyl-phenol, 2-hydroxy-3-methyl-2-cyclopenten-1-one, n-hexadecanoic acid, 2,3-dihydro-3,5-dihydroxy-6-methyl-4H-Pyran-4-one, and catechol (CAT) were all above 1%, while the contents of other compounds were relatively low. The volatile compounds in the crude extract of AG1-IB were divided into six main categories: phenols, carboxylic acids, esters, ketones, pyrimidines, and alkanes. The relative concentrations of benzeneacetic acid, 2-furancarboxylic acid, hydroquinone, diisooctyl phthalate, furyl hydroxymethyl ketone, and 4-hydroxybenzeneacetic acid were all above 1%. Only n-hexadecanoic acid was detected in both strains, and its content was six times greater in AG1-1A than in AG1-1B. These findings showed that there are great differences among the strains, which may be related to the pathogenic mechanisms of different AGs in the host. The eicosane content was the same as that in AG1-1A in healthy rice, and 2,2'-methylenebis[6-(1,1-dimethylethyl)-4-ethylphenol] had the same content as that found in AG1-1B, with higher content in healthy rice; notably, these compounds are not phytotoxins. A single compound was isolated and purified from the crude extract of AG1-IA by TLC, column chromatography, and HPLC. Its yellow crystal was characterized by NMR spectroscopy, and it had a molecular formula of C_6_H_6_O_4_. Spectral analysis revealed that the compound was 5-hydroxymethyl-2-furancarboxylic acid (HFA) (Munekata and Tamura, 1981). Combined with the findings of previous studies (Xuan et al., 2006; Kimura et al., 2007) and the differences in composition and content of crude extracts of AG1-IA and AG1-IB, we considered BZA, CAT, and HFA as candidate toxin components.

### 3.4 HPLC validation and activities of the three compounds

To verify the presence of candidate compounds in the leaf tissue of lettuce plants inoculated with the pathogen, we assessed the fermentation broth compositions of diseased spots and strains by HPLC. HFA was found in diseased lettuce leaves infected with M-9-11 and in the crude extracts of M-9-11 and HX-4C 2 days after inoculation. BZA was found in diseased lettuce leaves and in the isolate M-9-11, while CAT was found in only the fermentation product of strain M-9-11, which was presumed to not reach a detectable amount in pathogenic strains, possibly due to its easily oxidizable nature and other properties (**Table 1**).

**Table 1:** HPLC validation of the three compounds.

Sample	HFA	BZA	CAT
Diseased lettuce leaves inoculated by AG1-IA M-9-11	+	+	−
AG1-IA M-9-11	+	+	+
AG1-IB HX-4C	+	−	−

“+”, compound contained in the sample; “−“, compound not contained in the sample. HFA was found in diseased lettuce leaves 1 and 2 and in isolates M-9-11 and HX-4C 2 days after inoculation; BZA was found in diseased strains 1 and 2 and in M-9-11, while CAT was found in only the fermentation product of strain M-9-11.

**Figure 3:**
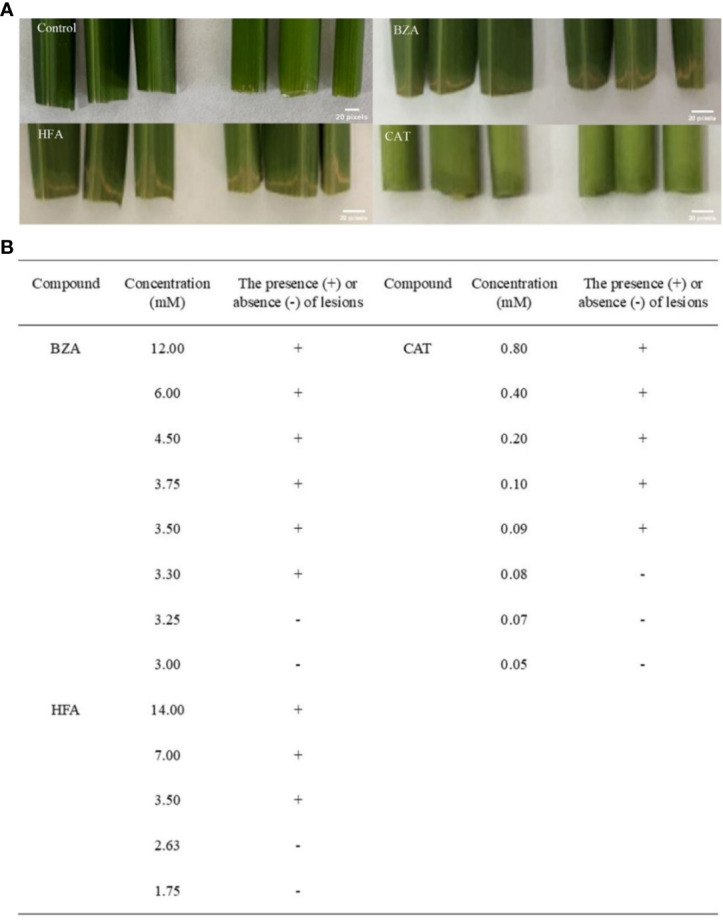
Determination of minimum activity concentrations for the three compounds. The rice tissue was mixed with each of the three compounds at the lowest concentrations that caused discoloration or necrosis of the rice tissue. **(A)** The phenomenon of treating rice with three compounds, **(B)** Determination of concentration of three compounds for treating rice.

To determine the minimum active concentrations of the three compounds, we performed precise quantitative tests by obtaining initial concentrations based on the relative concentrations detected by GC-MS and the concentrations of the compounds used for HPLC. According to the presence or absence of disease symptoms in rice tissues (**Figure 3**), the lowest concentrations of BZA, CAT, and HFA were 3.3 mM (400 ppm), 0.09 mM (10 ppm), and 3.5 mM (500 ppm), respectively. BZA and HFA caused yellow necrosis in rice leaves and leaf sheaths, while CAT caused black necrosis in rice leaf sheaths. These three compounds fulfill the conditions outlined in the review of functional phytotoxins: they cause host plant cell death or suppress the vital activity of plant cells at concentrations less than 10 mmol (Berestetskiy, 2008).

### 3.5 Detect whether the three compounds affect membrane lipid components

**Figure 4:**
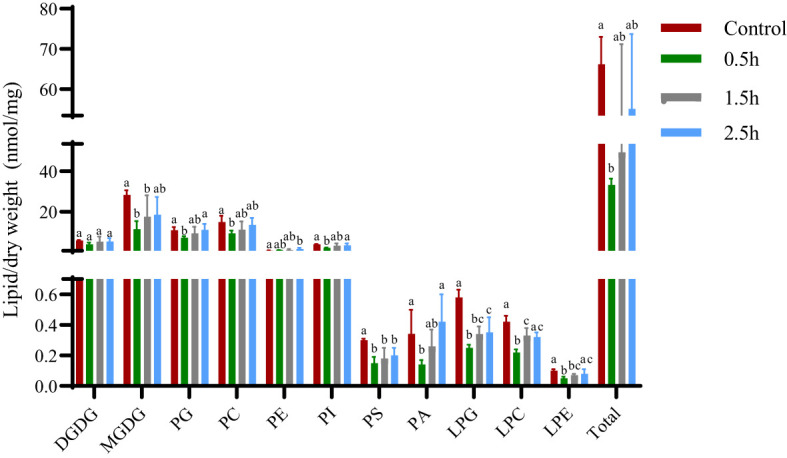
Changes in membrane lipid entities and total lipids of *A. thaliana* after treatment with crude extract. Five replicates of each sample were analyzed. The Tukey**’**s HSD test (DPS v9.01) revealed pairwise significant differences at *p***<** 0.05, represented by the lowercase letters.

The cell membrane is an important barrier for plant cells to communicate with external organisms, and the environment is also the basis for ensuring that all antioxidant mechanisms within the cell are normal. With the emergence of unfavorable factors in the environment, the lipid and fatty acid components of the cell membrane, which are the main carbon chain length and unstable saturation components, cause the cell membrane to undergo substantial changes to adapt to external changes (Shevchenko and Simons, 2010). Changes in lipid entities and total lipids in *A. thaliana* plants treated with crude extract showed (**Figure 4**) that, except for PE, the contents of other membrane lipids first decreased and then increased, and different changes were observed for the small molecules of each lipid. After crude extract treatment, the PE content increased continuously, with a significant difference at 2.5 h compared with the control value. The contents of MGDG, PG, PC, PI, PS, PA, LPG, LPC, and LPE were lowest at 0.5 h, significantly lower than the control values, and then increased; of these, the contents of the lysophospholipids LPG and LPC were significantly lower than control values at three time points. The lysophospholipid LPE concentration significantly differed between 0.5 h and 1.5 h. Although the DGDG content decreased at 0.5 h, there was no significant difference in content among the treatments; the changes in total lipids were similar to those in lipid entities. At 0.5 h, the total membrane lipid content in *A. thaliana* was significantly lower than that in the control plants and subsequently increased. These results indicated that the toxin did not directly damage membrane lipid molecules.

### 3.6 Effect of the three compounds on the structural integrity of the membrane

**Figure 5:**
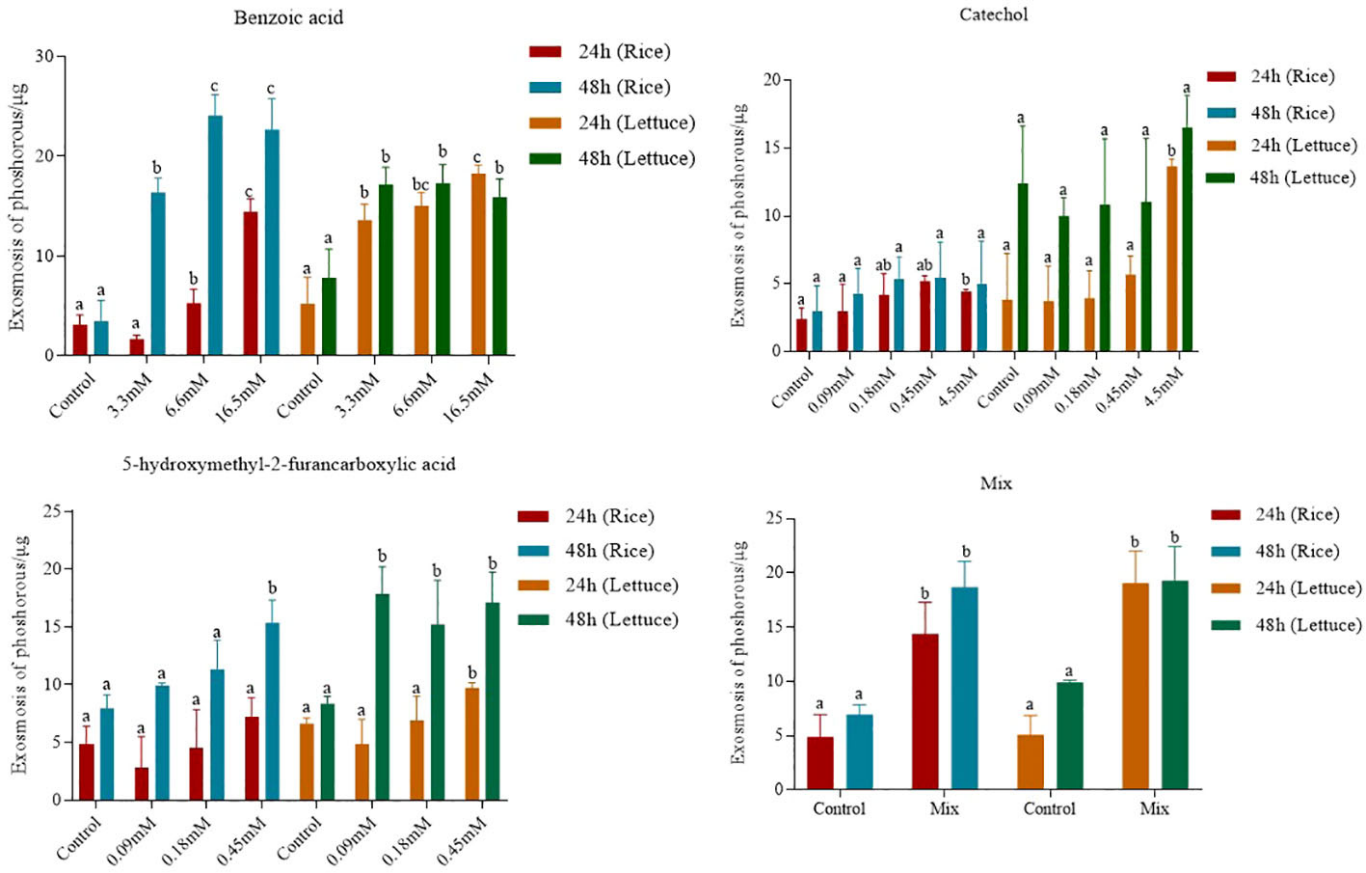
Phosphorus extravasation in rice leaves after treatment with three compounds. Five replicates were analyzed for each sample. Tukey’s HSD test (DPS v9.01) revealed pairwise significant differences at *p*< 0.05, represented by the lowercase letters.

**Figure 6:**
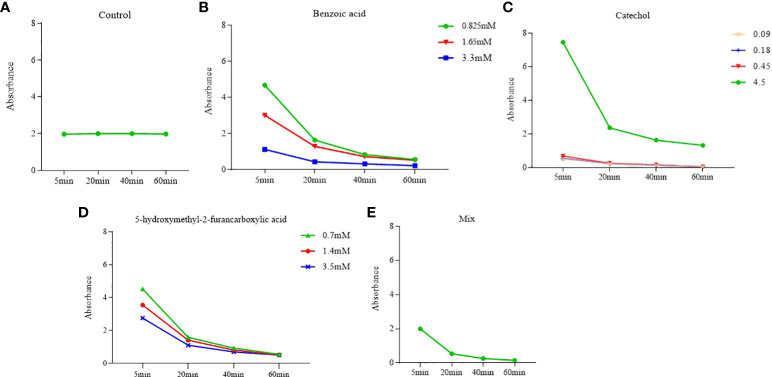
Effects of phytotoxins on membrane hydrophobicity. Five replicates were analyzed for each sample. BZA and HFA mixed with all three compounds induced a faster reduction in membrane hydrophobicity at low concentrations, while CAT caused a faster reduction in membrane hydrophobicity only at high concentrations. **(A)** Control, **(B)** Benzoic acid, **(C)** Catechol, **(D)** 5-hydroxymethyl-2-furancarboxylic acid, **(E)** Mix.

**Figure 7:**
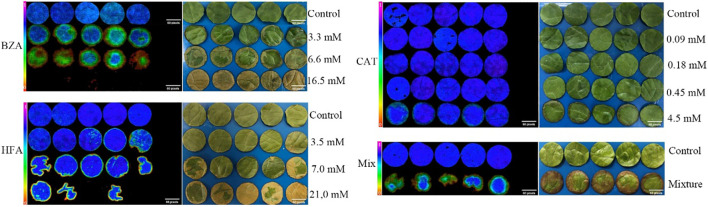
The Fv/Fm and phenotype of lettuce after treatment with three compounds. Five replicates were analyzed for each sample. The mixture contained 3.3 mM BZA, 3.5 mM HFA, and 0.09 mM CAT.

**Figure 8:**
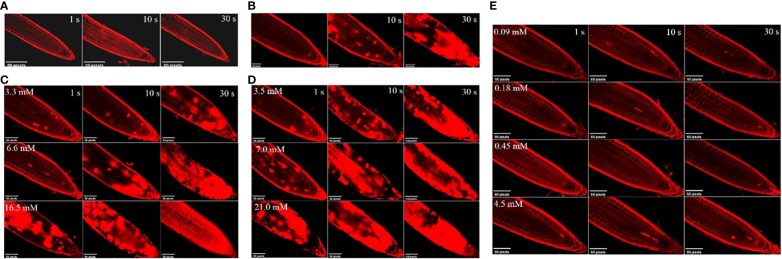
Effects of different concentrations of the test compounds on young roots of *A. thaliana* at various treatment times. **(A)** Control. **(B)** The three compounds were mixed at the lowest concentration (BZA: 3.3 mM; HFA: 3.5 mM; CAT: 0.09 mM) to form a mixture. After acting on the roots of *A. thaliana* for 1 s, 10 s, and 30 s, the nucleus of *A. thaliana* root tip was stained with PI dye. **(C)** 3.3 mM, 6.6 mM, or 16.5 mM BZA was applied to *A. thaliana* roots; after 1 s, 10 s, or 30 s, Arabidopsis root tip nuclei were stained with PI dye. **(D)** 3.5 mM, 7.0 mM, or 21.0 mM HFA was applied to *A. thaliana* roots; after 1 s, 10 s, and 30 s, *A. thaliana* root tip nuclei were stained with PI dye. **(E)** 0.09 mM, 0.18 mM, 0.45 mM, or 4.5 mM CAT was applied to *Arabidopsis* roots; after 1 s, 10 s, or 30 s, *A. thaliana* root tip nuclei were stained with PI dye.

**Figure 9:**
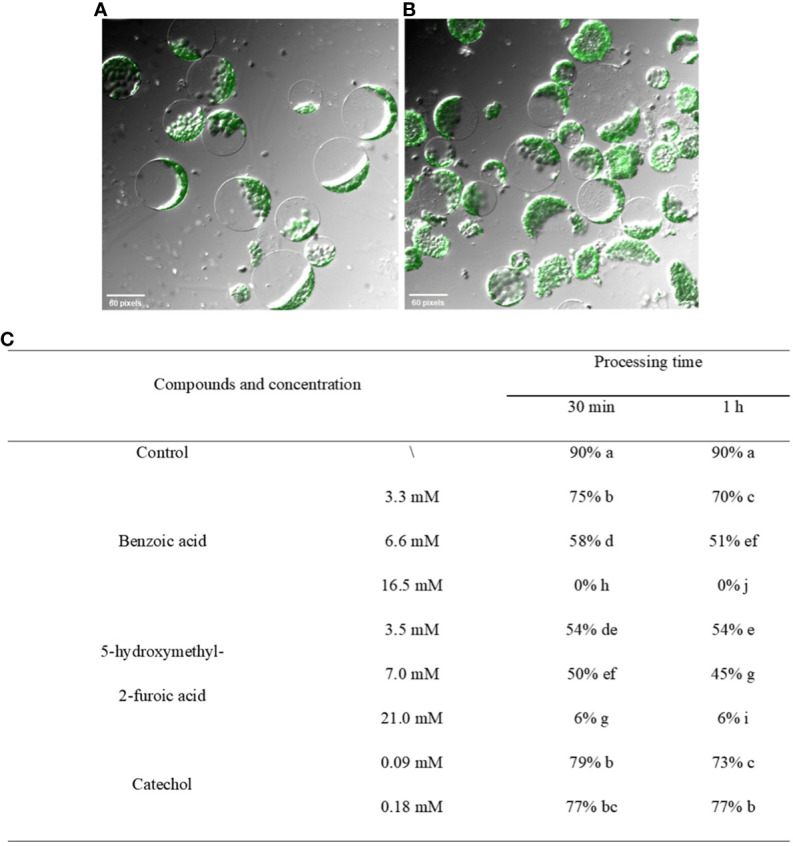
Effects of crude extract on protoplasts of *A. thaliana*. **(A)** Untreated protoplast morphology. **(B)** Morphological changes in protoplasts after the addition of the three compounds to liquid medium containing protoplasts. **(C)** After treatment with different concentrations of the test compounds, the percentage of normal plants was counted, and the results were found to decrease in all the treatment groups in a manner inversely proportional to the concentration and treatment time. Five replicates were analyzed for each sample. The Tukey’s HSD test (DPS v9.01) revealed pairwise significant differences at *p*< 0.05, represented by the lowercase letters.

The effects of the three compounds on the integrity of the plant cell membrane were verified by the following groups of experiments. The amount of phosphorus extravasation in plant tissues increased after the treatment of crude extract, indicating that the cell membrane system of plant tissues was destroyed (Zuo et al., 2014). Therefore, we first evaluated the effects of these compounds on phosphorus extravasation in rice leaves. The results (**Figure 5**) showed that 3.3 mM BZA and 21 mM HFA significantly induced phosphorus extravasation in rice leaves after treatment with the above compounds for 48 h. No effect of CAT on phosphorus extravasation was observed. The phospholipid bilayer in the biofilm structure is divided into hydrophilic and hydrophobic ends. We mix water and oil and ultrasonically treated them to form emulsions that rearrange the oil droplets in the water. After the compounds were added to the emulsion, the results showed that 3.3 mM BZA, 0.09 mM CAT, and 3.5 mM HFA reduced absorbance within 5 min. These results showed that all three compounds could quickly destroy hydrophobic forces maintaining the membrane structure (**Figure 6**). Fv/Fm, a chlorophyll fluorescence index, is used to characterize the conversion efficiency of light energy in the PS II reaction center. Fv/Fm diagrams for different concentrations of BZA, CAT, and HFA as well as the mixture of these three compounds at low concentrations after treatment for 12 h revealed damaged leaves. As shown in **Figure 7**, BZA and HFA caused symptoms in leaves at 3.3 mM and 3.5 mM, respectively, while CAT caused symptoms in leaves at 4.5 mM. Subsequently, we treated the roots of Arabidopsis seedlings with crude extracts and stained them with propidium iodide (PI). The results revealed almost completely stained nuclei in Arabidopsis root tip cells after treatment for 30 s with 16.5 mM BZA, 3.5 mM HFA, or 21 mM CAT. With the prolongation treatment time and increasing concentration, the number of PI-stained Arabidopsis root tip nuclei gradually increased (**Figure 8**). Protoplasts are living naked cells surrounded by a plasma membrane after removal of the cell wall, facilitating direct observation of the effects of crude extract on the cell membrane. Therefore, we added crude extract substances to the protoplasts and observed the changes. The proportions of protoplasts with a normal form decreased after treatment with different concentrations of the test compounds and were inversely proportional to the compound concentrations and treatment times (**Figure 9**). The above results confirmed the damaging effects of the three compounds on the integrity of the plant cell membrane.

### 3.7 Effects of the three compounds on cell structure and organelles

**Figure 10:**
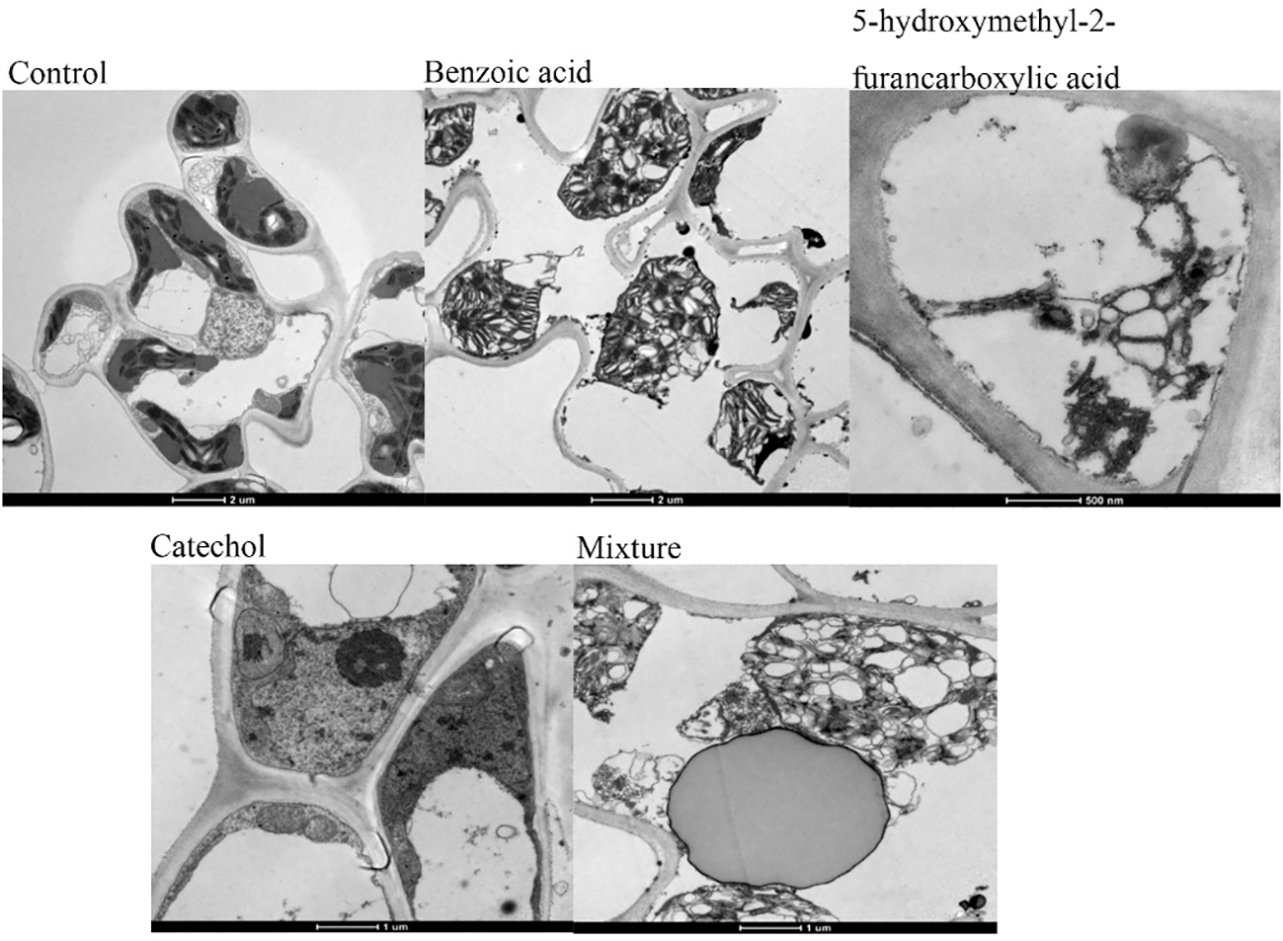
Ultrastructure of rice leaf cells. Treatment of rice leaves with all three compounds, separately or in combination, induced changes in the ultrastructure of the leaves, including plasma wall separation, small vesicles around the cytoplasmic membrane, breakages, irregular mitochondrial morphology, bilayer membrane disruption, increased starch grains, partial enlargement, chloroplast swelling and disintegration, and chromatin agglutination.

We assessed whether the three compounds continued to affect various organelles within the membrane after damaging the cell membrane. The results (**Figure 10**) showed that three components not only damaged the plasma membrane but also functioned at the subcellular level, destroying the membrane structures of other organelles, as observed by electron microscopy. After treatment with 3.3 mM BZA for 12 h, chloroplast swelling and disintegration and mitochondrial crista were observed, as was the double-layer membrane, nucleolus, and nuclear membrane disappearance, with multiple vesicles appearing and increasing amounts of starch granules. After treatment with 16.5 mM BZA, cytoplasmic wall separation, cytoplasmic membrane breakage, irregular chloroplast morphology, partial chloroplast disintegration and vacuolization, partial chloroplast shrinkage, internal structure condensation, grana formation, matrix disintegration, mitochondrial bilayer membrane disappearance, nucleus malformation, and increased starch granule formation were induced. These results indicated that these three components not only destroy the plasma membrane but also damage the membrane structures of other organelles in host cells.

## 4 Discussion

Currently, the main pathogenic factors of *R. solani* are toxins and cell wall-degrading enzymes, but different opinions exist regarding the toxin components in *R. solani*. One of the main areas of future research will be to identify one toxin component among the metabolites and examine its molecular structure. A quantitative analysis of the toxin’s biological activity is also needed (Xu et al., 2020). To date, most of the toxin components of *R. solani* are considered carboxylic acids and their derivatives or sugars and sugar amines. Previously, Aoki and Sassa (1963) isolated two strains of *R. solani* from sugar beet, and their metabolites were lactic acid, succinic acid, phenylacetic acid, 2-furancarboxylic acid, and m-hydroxyphenylacetic acid. Mandava et al. (1980) isolated AG1-IA subgroup strains from soybeans. The metabolites were carboxylic acids and derivatives, and a new compound, m-methoxyphenylacetic acid, was found for the first time. The content of carboxylic acids and their derivatives was also quantitatively analyzed by HPLC. Subsequently, Xu et al. (2015) reported that methyl 3-hydroxyphenylacetate, (Z)-3-methylpent-2-en-1,5-dicarboxylic acid, and 3-methoxyfuro-2-carboxylic acid were identified from *R. solani* isolated from rice. Hu et al. (2017) first identified tenuazonic acid in AG1-IA. On the other hand, Sriram et al. (2000) reported that *R. solani* toxin is a mixture of sugars and glycosamines containing glucose, mannose, and N-acetylgalactosamine. Chen et al. (2011) detected glucose, sucrose, and N-acetylaminomannose. Yang et al. (2011) detected several carbohydrates. To date, the determination of the main toxic components of *R. solani* and whether and how they work together need to be further studied. In this study, GC-MS and other techniques were used to identify the toxin components in the crude extract of *R. solani* AG1-IA, such as BZA, CAT, and HFA, and these techniques were combined with HPLC to quantitatively study the toxins.

Destroying the membrane system is an important feature of deadly parasites. Pathogenic mycotoxins aimed at ensuring membrane integrity, including sphingosine, AAL-toxin, fumonisin, cyperin, and deticolin 0, have been reported (Marcel et al., 2010). Previous studies by Chen et al. (2009) showed that electrolyte leakage was significant after *R. solani* toxin treatment of rice tissues, and the longer the treatment time was, the greater the toxin concentration and the greater the leakage rate. This is because toxins act on lipid and protein biofilms, resulting in changes in cell membrane permeability and electrolyte leakage. In this study, we first determined the contents of 11 membrane lipid molecules in plant leaves treated with crude extract by ESI-MS/MS. The molecular contents of plant membrane lipids decreased first after treatment with crude extract before increasing to control levels. The results showed that the crude extract did not degrade membrane lipids or alter membrane lipid metabolism but can change the state and permeability of membrane lipids while maintaining the same membrane composition. After BZA and HFA treatment, the amount of phosphorus extravasation in plant leaves increased significantly, which confirmed that the selective permeability of the plant cell membrane changed, indicating that the cell membrane of the plant tissue was destroyed after treatment. It also caused changes in photosynthetic parameters, indicating that the chloroplast membrane structure in plant tissues was disrupted and that the electron transfer rate decreased, confirming that the thylakoid membrane structure was destroyed. The nucleus of Arabidopsis root tip was stained with the fluorescent dye, which indicated that the cell membrane of the Arabidopsis root tip was destroyed. The results of Arabidopsis protoplast distortion and chloroplast grana swelling also showed that the cell membrane was destroyed. The above results confirmed the damaging effects of BZA and HFA to the integrity of the plant cell membrane. In addition, the activity of BZA was similar to that of the crude extract. Therefore, we believe that BZA is one of the main components of *R. solani* toxin. For pyrocatechol, 0.09 mM CAT could hardly cause phosphorus leakage in plant tissues within 24 h, nor could it cause changes in photosynthetic parameters, and had little effect on hydrophobic force. The degree of destruction of protoplasts was also low, and the cell membrane of the Arabidopsis roots was not destroyed within the range of experimental conditions. However, in the *in vitro* activity test, 0.09 mM CAT could cause black lesions in the rice sheath within 24 h. Therefore, we speculated that CAT was pathogenic to plant tissues, but that its main site of action may not be the membrane system of plant tissues.

Changes in cell ultrastructure can reflect the pathogenic sites and pathogenic mechanisms of toxins to a certain extent. Previous studies have shown that different mycotoxins have different action sites in cells. For example, the main site of action of AK toxin is the plasma membrane (Yang et al., 2017), the main site of action of ACR toxin is the mitochondria (Kodama et al., 2008), and the main site of action of corn T toxin is the mitochondria (Matthews et al., 2017). Chen et al. (2009) and Kankam et al. (2016b) studied cells treated with *R. solani* crude extract solution, and reported that the cell structure was significantly damaged. Hu et al. (2018) showed that tenuazonic acid, identified in the crude extract of *R. solani*, is a natural phytotoxin and has been proven to be a new type of PS II inhibitor. In the present study, the cell ultrastructures of rice leaves treated with three toxin compounds and their mixtures were observed. After the treatment of rice leaves with BZA and HFA, the chloroplasts first disintegrated, while some mitochondria disintegrated. Although the double membrane and ridge of some mitochondria became blurred, the degree of damage was less than that of chloroplasts, and the rupture of the plasma membrane also occurred after the disintegration of chloroplasts. The changes in chloroplast caused by CAT were also greater than the changes in mitochondria. Therefore, we believe that the cell structure of these three compounds target is chloroplasts.

The crude extract components of the weak pathogenic fungus AG1-IB were detected. The results showed that the crude extract of the AG1-IB strain did not contain BZA or CAT, but contained a certain amount of HFA, and these three compounds were not detected in the crude extract of healthy rice sheath. Therefore, we believe that the difference in pathogenicity between AG1-IA and AG1-IB is mainly related to the two chemicals used (BZA and CAT). Both the crude extracts of the diseased plants and the strains contained BZA and HFA, but the HPLC results of the diseased plants showed that there was no CAT, and the fermentation products of the strains contained a large amount of CAT. Therefore, we speculate that the *R. solani* strain itself will metabolize CAT, but it may be oxidized after acting on the plant, so that CAT is not detected in the extract of the diseased plant.

In conclusion, we screened and identified three AG1-IA toxin components: BZA, HFA, and CAT. Moreover, these components can rapidly disintegrate the biofilm structure by maintaining the content of membrane components, affecting the normal physiological function of the membrane structure, and subsequently attacking organelles, especially chloroplasts, causing irreversible damage to plants, which may also be the reason for the lack of highly resistant varieties of cultivated rice.

## Data availability statement

The original contributions presented in the study are publicly available. This data can be found here: https://figshare.com/s/0289b4ca337c1b149bf6.

## Author contributions

SX: Data curation, Writing – review & editing. SR: Methodology, Writing – original draft. WB: Methodology, Writing – original draft. XL: Software, Writing – original draft. YZ: Methodology, Writing – original draft. BY: Methodology, Writing – original draft. WL: Methodology, Writing – original draft. CL: Funding acquisition, Supervision, Writing – review & editing. WD: Formal analysis, Supervision, Writing – original draft. GY: Conceptualization, Funding acquisition, Supervision, Writing – review & editing.
