# Supplementary material for: Identification of the toxin components of Rhizoctonia solani AG1-IA and its destructive effect on plant cell membrane structure
Source: Front Plant Sci. 2024 Feb 13;15:1348257. doi: 10.3389/fpls.2024.1348257 (PMC10896845; doi:10.3389/fpls.2024.1348257)
Supplement: Supplementary file 1 [file Table_1.doc]

**Screening of *Rhizoctonia solani* AG1-IA effector proteins and toxins and disruptive effects of toxin components on plant cell membrane structure**

Shanshan Xu ^1*^, Shaofeng Ren^1*^, Wenjing Bao^1^, Xiaoguang Li^1^, Yumei Zhang^2^, Buzhu Yu ^3^, Weiqi Li ^3^, Chengyun Li ^1^, Wenhan Dong^1^, Genhua Yang^1^

^1^ State Key Laboratory for Protection and Utilization of Bio-Resources in Yunnan, Yunnan Agricultural University, Kunming 650201, Yunnan, China. ^2^ Xishuangbanna Tropical Botanical Garden, Chinese Academy of Sciences. ^3^ Institute of Botany, Chinese Academy of Sciences

**Supplementary Table S1 Volatile chemical constituents of AG1-IA’s crude extract.** The following compounds were detected by GC-MS with similarity greater than 85%.

| S/n | Name of compound | Matching degree (%) | Relative content (%) | Molecular formula |
| --- | --- | --- | --- | --- |
| 1 | Eicosyl acetate | 97 | 0.27 | C_22_H_44_O_2_ |
| 2 | Benzoic acid | 96 | 6.44 | C_7_H_6_0_2_ |
| 3 | 2,2'-methylenebis-6-(1,1-dimethylethyl)-4-methyl- Phenol | 96 | 7.6 | C_23_H_32_O_2_ |
| 4 | 2-hydroxy-3-methyl-2-cyclopenten-1-one | 95 | 3.83 | C_6_H_8_O_2_ |
| 5 | n-Hexadecanoic acid | 95 | 1.56 | C_16_H_32_O_2_ |
| 6 | Heneicosane | 95 | 0.16 | C_21_H_44_ |
| 7 | Octadecanoic acid | 95 | 0.65 | C_18_H_36_O_2_ |
| 8 | Bis(2-ethylhexyl) phthalate | 93 | 0.38 | C_24_H_38_O_4_ |
| 9 | 2,3-dihydro-3,5-dihydroxy-6-methyl-4H-pyran-4-one | 91 | 14.37 | C_6_H_8_O_4_ |
| 10 | 2-Propenoic acid, pentadecyl ester | 91 | 0.41 | C_18_H_34_O_2_ |
| 11 | 2-cyclohexylidene-cyclohexanone | 90 | 0.81 | C_12_H_18_O |
| 12 | N, N-dimethyl-octanamide | 89 | 0.16 | C_10_H_21_NO |
| 13 | 2,3-dimethyl-3-Hexanol | 88 | 0.63 | C_8_H_18_O |
| 14 | N, N-Dimethyl-dodecanamide | 88 | 0.08 | C_14_H_29_NO |
| 15 | Catechol | 87 | 4.68 | C_6_H_6_O_2_ |
| 16 | Eicosane | 85 | 0.08 | C_20_H_42_ |

**Supplementary Table** **S2 Volatile chemical constituents of AG1-IB’s crude extract.** The following compounds were detected by GC-MS with similarity greater than 85%.

| S/n | Name of compound | Matching degree (%) | Relative content (%) | Molecular formula |
| --- | --- | --- | --- | --- |
| 1 | Benzeneacetic acid | 98 | 6.27 | C_8_H_8_O_2_ |
| 2 | 2-Furancarboxylic acid | 96 | 4.37 | C_5_H_4_O_3_ |
| 3 | Hydroquinone | 96 | 6.51 | C_6_H_6_O_2_ |
| 4 | Phenol, 2,2'-methylenebis[6-(1,1-dimethylethyl)-4-methyl- | 96 | 0.21 | C_23_H_32_O_2_ |
| 5 | Diisooctyl phthalate | 96 | 3.78 | C_24_H_38_O_4_ |
| 6 | n-Hexadecanoic acid | 95 | 0.25 | C_16_H_32_O_2_ |
| 7 | Furyl hydroxymethyl ketone | 94 | 2.46 | C_6_H_6_O_3_ |
| 8 | Octadecanoic acid | 92 | 0.08 | C_18_H_36_O_2_ |
| 9 | 2,5-Furandione, dihydro-3-methylene- | 91 | 0.95 | C_5_H_4_O_3_ |
| 10 | Benzoic acid, 4-hydroxy- | 91 | 0.94 | C_7_H_6_O_3_ |
| 11 | Benzeneacetic acid, 4-hydroxy- | 91 | 3.48 | C_8_H_8_O_3_ |
| 12 | 6-Azacytosine | 86 | 0.58 | C_3_H_4_N_4_O |

**Supplementary Table S3 Volatile chemical constituents of health rice crude extract.** The following compounds were detected by GC-MS with similarity greater than 85%.

| S/n | Name of compound | Matching degree (%) | Relative content (%) | Molecular formula |
| --- | --- | --- | --- | --- |
| 1 | Hexadecanoic acid, methyl ester | 97 | 29.97 | C_17_H_34_O_2_ |
| 2 | 9,12-Octadecadienoic acid (Z,Z)-, methyl ester | 96 | 25.37 | C_19_H_34_O_2_ |
| 3 | Methyl stearate | 96 | 1.59 | C_19_H_38_O_2_ |
| 4 | Bis(2-ethylhexyl) phthalate | 96 | 1.19 | C_24_H_38_O_4_ |
| 5 | Cycloheptasiloxane, tetradecamethyl- | 95 | 1.73 | C_14_H_42_O_7_ |
| 6 | Heptadecanoic acid, methyl ester | 94 | 0.73 | C_18_H36O2 |
| 7 | Eicosane | 93 | 0.66 | C_20_H_42_ |
| 8 | Phenol,2,2’-methylenebis[6-(1,1-dimethylethyl)-4-methyl- | 93 | 1.47 | C_23_H_32_O_2_ |
| 9 | Cyclononasiloxane, octadecamethyl- | 91 | 1.1 | C_18_H_54_O_9_ |
| 10 | 11,14,17-Eicosatrienoic acid, methyl ester | 91 | 21.47 | C_21_H_36_O_2_ |
| 11 | Cyclooctasiloxane, hexadecamethyl- | 86 | 0.89 | C_16_H_48_O_8_ |
| 12 | Hexatriacontane | 91 | 0.21 | C_36_H_74_ |
| 13 | Methyl 18-methylnonadecanoate | 89 | 0.21 | C_21_H_42_O_2_ |
| 14 | Cyclodecasiloxane, eicosamethyl- | 85 | 0.65 | C_20_H_60_O_1_ |
